# Worthing Physiological Score vs Revised Trauma Score in Outcome Prediction of Trauma patients; a Comparative Study 

**Published:** 2017-01-11

**Authors:** Babak Nakhjavan-Shahraki, Mahmoud Yousefifard, Mohammad Javad Hajighanbari, Parviz Karimi, Masoud Baikpour, Jalaledin Mirzay Razaz, Mehdi Yaseri, Kavous Shahsavari, Fatemeh Mahdizadeh, Mostafa Hosseini

**Affiliations:** 1 Sina Trauma and Surgery Research Center, Tehran University of Medical Sciences, Tehran, Iran.; 2 Physiology Research Center and Department of Physiology, Faculty of Medicine, Iran University of Medical Sciences, Tehran, Iran.; 3 Department of Emergency Medicine, Hafte Tir Hospital, Iran University of Medical Sciences, Tehran, Iran.; 4 Department of Emergency Medicine, Robatkarim Hospital, Iran University of Medical Sciences, Tehran, Iran.; 5 Department of Medicine, School of Medicine, Tehran University of Medical Sciences, Tehran, Iran.; 6 Department of Community Nutrition, Faculty of Nutrition and Food Technology, Shahid Beheshti University of Medical Sciences, Tehran, Iran.; 7 Department of Epidemiology and Biostatistics, School of Public Health, Tehran University of Medical Sciences, Tehran, Iran.; 8 Road Traffic Injury Research Center, Tabriz University of Medical Sciences, Tabriz, Iran.; 9 Department of Emergency Medicine, Ilan University of Medical Sciences, Ilam, Iran.

**Keywords:** Trauma Severity Indices, Prognosis, Trauma, emergency department, decision support techniques

## Abstract

**Introduction:**

Awareness about the outcome of trauma patients in the emergency department (ED) has become a topic of interest. Accordingly, the present study aimed to compare the rapid trauma score (RTS) and worthing physiological scoring system (WPSS) in predicting in-hospital mortality and poor outcome of trauma patients.

**Methods:**

In this comparative study trauma patients brought to five EDs in different cities of Iran during the year 2016 were included. After data collection, discriminatory power and calibration of the models were assessed and compared using STATA 11.

**Results:**

2148 patients with the mean age of 39.50±17.27 years were included (75.56% males). The AUC of RTS and WPSS models for prediction of mortality were 0.86 (95% CI: 0.82-0.90) and 0.91 (95% CI: 0.87-0.94), respectively (p=0.006). RTS had a sensitivity of 71.54 (95% CI: 62.59-79.13) and a specificity of 97.38 (95% CI: 96.56-98.01) in prediction of mortality. These measures for the WPSS were 87.80 (95% CI: 80.38-92.78) and 83.45 (95% CI: 81.75-85.04), respectively. The AUC of RTS and WPSS in predicting poor outcome were 0.81 (95% CI: 0.77-0.85) and 0.89 (95% CI: 0.85-0.92), respectively (p<0.0001).

**Conclusion:**

The findings showed a higher prognostic value for the WPSS model in predicting mortality and severe disabilities in trauma patients compared to the RTS model. Both models had good overall performance in prediction of mortality and poor outcome.

## Introduction

Quick assessment of trauma patients and knowledge about the severity of their injuries can significantly affect the outcome of these patients, decrease their mortality rates and their associated disabilities (-). Awareness about the final outcome of trauma patients in the emergency setting has become a topic of discussion in recent years and various methods have been proposed to address this issue. In this regard, different scoring systems have been developed (-). Over the years these scoring systems became so popular among physicians that encouraged further development of these models. Application of these scoring systems help in identifying high-risk patients (9), which leads to a better controlled management and treatment of patients. Nevertheless, each of these scoring systems have their own shortcomings, some of which include numerous variables involved in the model, complicated calculations needed to reach a conclusion (e.g. injury severity score) and their validity and reliability not having been assessed in different clinical settings. These limitation encouraged researchers to design better systems, the examples of which are the revised trauma score (RTS), rapid acute physiology score (RAPS), rapid emergency medicine score (REMS) and Worthing Physiological Scoring System (WPSS) (-). 

RTS is a scoring system based on physiologic variables of Glasgow coma scale (GCS), systolic blood pressure (SBP) and respiratory rate (RR), in which the GCS has higher weight compared to the other two variables. However, its low prognostic value for outcome of trauma patients pushed the researchers to search for other scoring systems (12, 14). 

WPSS was another scoring system presented in the year 2007. The model was designed based on a study conducted on 3184 patients that found the 6 factors of RR, pulse rate, SBP, body temperature, the oxygen saturation and the level of consciousness assessed on arrival of the patients to be able to predict their mortality (11). However, little information is available on the overall validity of this model. Accordingly, the present study was designed to assess and compared the value of WPSS and RTS models in prediction of in-hospital mortality and poor outcome in trauma patients presenting to the emergency departments. 

## Methods

***Study design and setting***

In this prospective cross-sectional study, trauma patients brought to five emergency departments in different cities of Iran (Tehran, Ilam, Jahrom, Tabriz and Urmia) from May to October 2016 were included. Completed checklists were posted to Tehran and reviewed by the senior researcher. After verifying their validity, gathered data were analyzed using the statistical software. The Ethics Committee of Tehran University of Medical Sciences reviewed and approved the study protocol. The guidelines laid down by Declaration of Helsinki were adhered to by all the authors throughout the survey and all the included patients or their family members signed an informed written consent for participating in the study. 

***Participants***

Trauma patients older than 18 years of age brought to the designated emergency departments were included as the study population through a convenience sampling method. Pregnancy and death before admission to the emergency department were considered as the exclusion criteria. 

***Data gathering***

Gathered information included age, gender, trauma mechanism, vital signs, arterial oxygen saturation level, and level of consciousness on admission. The patients were followed throughout their hospital stay and their final outcome (expired vs. alive) along with the condition in which the patient was discharged from the hospital (full recovery, moderate disability, severe disability or vegetative state) were recorded. 

***Assessed outcomes***

Glasgow outcome scale (GOS) was used to assess the final outcome of the patient when being discharged from the hospital (20). In-hospital mortality was considered as the primary outcome and discharge with a severe disability (based on GOS) was considered as the secondary outcome. 

***Statistical analysis***

In order to calculate the minimum sample size needed for this survey, the rate of in-hospital mortality in trauma patients was considered as 5.2% based on previous reports (21). Accordingly, the minimum sample size was estimated at 1894 patients based on a 95% confidence interval (CI) (α=0.05), a 90% power (β=0.1) and a maximum error of 1.5% (d=0.015). 

Data analysis was performed by STATA 11.0 software. Severity of trauma were calculated for each patient based on RTS and WPSS models and the prognostic value of the systems was compared according to the discrimination power, calibration and overall performance. 

Discrimination was evaluated by measuring the area under the curve (AUC) of the receiver operating characteristic (ROC) curve and calculating the sensitivity, specificity, positive and negative likelihood ratios with 95% CI. The method proposed by Cleves and Rick was used for comparing AUC for the two models (22). 

Calibration plot was constructed for assessment of general calibration in which the frequency of observed versus predicted mortality or poor outcome were compared. Overall performance was assessed by evaluating the predictive reliability and predictive accuracy based on the calculated Brier score. Finally, in order to assess the concordance between RTS-predicted and WPSS-predicted percent of mortality and poor outcome, Spearman’s rank coefficient was computed. A p value less than 0.05 was considered as statistically significance level in all analyses. 

## Results

A total of 2148 patients with the mean age of 39.50±17.27 year were included in this survey (75.56% male). Motorcycle accident was the most common trauma mechanism (75.65%). GCS ranged from 3-8 in 63 patients (2.98%), 9-12 in 36 patients (1.7%) and it was higher than 13 in 2014 cases (95.3%). Table 1 presents the basic characteristics of the studied subjects. Follow up of the subjects revealed that only 2.47% of the patients were discharged with severe disabilities and 5.73% of the cases expired. 

***Performance of RTS and WPSS in prediction of mortality***

***Discrimination***

**Figure 1:**
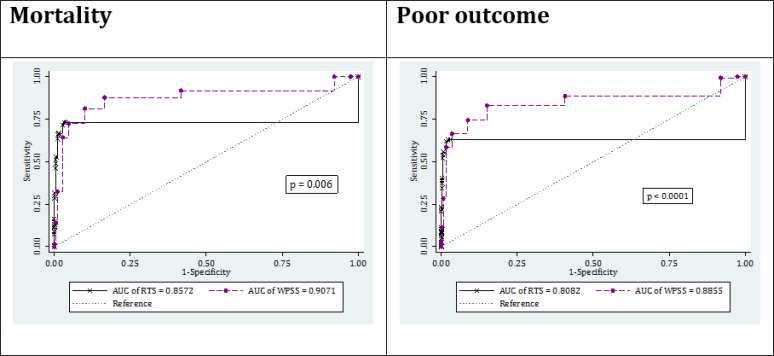
Area under the curve (AUC) of revised trauma score (RTS) and worthing physiological scoring system (WPSS) in prediction of in-hospital mortality and poor outcome

The AUC of the two RTS and WPSS models for prediction of patients’ mortality was calculated to be 0.86 (95% CI: 0.82-0.90) and 0.91 (95% CI: 0.87-0.94), respectively (p=0.006). The optimum cut-off level was found to be 1 for the RTS and 4 for the WPSS. The sensitivity and specificity of the RTS model for predicting patients’ mortality was calculated to be 71.54 (95% CI: 62.59-79.13) and 97.38 (95% CI: 96.56-98.01), respectively. These measures for the WPSS were found to be 87.80 (95% CI: 80.38-92.78) and 83.45 (95% CI: 81.75-85.04), respectively (Figure 1 and Table 2). 

***Calibration***

**Figure 2:**
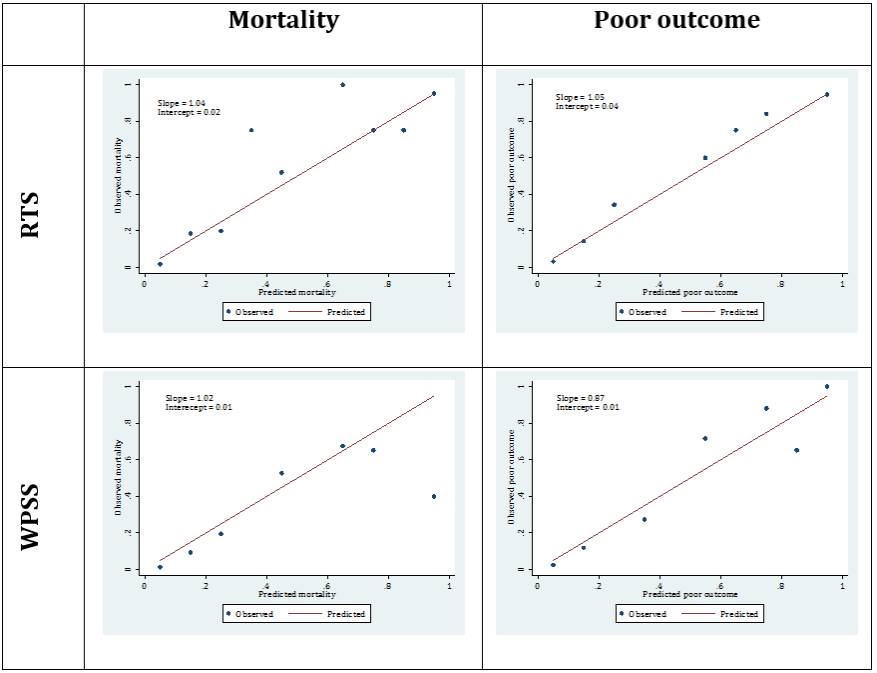
Calibration plots of revised trauma score (RTS) and worthing physiological scoring system (WPSS) in prediction of in-hospital mortality and poor outcome

Both scoring systems had good calibration (agreement between observed and predicted rate of mortality) in prediction of mortality. Calibration plot of the RTS model had a slope of 1.04 and an intercept of 0.02. The mentioned measured were calculated to be 1.02 and 0.01 for the WPSS model, respectively (Figure 2). 

***Overall performance***

Brier score and scaled reliability of the RTS model in prediction of mortality were 0.024 and zero, respectively. These measures were found to be 0.031 and 0.0003 for the WPSS model, respectively. The findings exhibit the high predictive accuracy and reliability of both models (Table 3). 

**Performance of RTS and WPSS in prediction of poor outcome**

***Discrimination***

The RTS model had an AUC of 0.81 (95% CI: 0.77-0.85) in predicting poor outcome, which was significantly lower than that of WPSS model with an AUC of 0.89 (95% CI: 0.85-0.92) (p<0.0001). The sensitivity and specificity of the RTS model for predicting poor outcome was found to be 61.93 (95% CI: 54.29-69.05) and 98.38 (95% CI: 97.69-98.87) considering the cut-off value of 1, respectively. These figures for the WPSS model with a cut-off level of 4 were calculated to be 82.95 (95% CI: 76.40-88.03) and 84.95 (95% CI: 83.27-86.47), respectively (Figure 1 and Table 2). 

***Calibration***

Both scoring systems had good calibration in predicting poor outcome of patients as well. The slope and intercept of the RTS model’s calibration plot were 1.05 and 0.04, respectively. The mentioned measures were 0.87 and 0.01 for the WPSS model’s calibration plot (Figure 2). 

***Overall performance***

Brier score and scaled reliability calculated for RTS model in predicting patients’ poor outcome were 0.034 and zero, while these measures were found to be 0.045 and 0.001 for the WPSS model, respectively (Table 3). Both RTS and WPSS models have good overall performance in prediction of poor outcome. 

**Concordance between RTS and WPSS**

**Figure 3:**
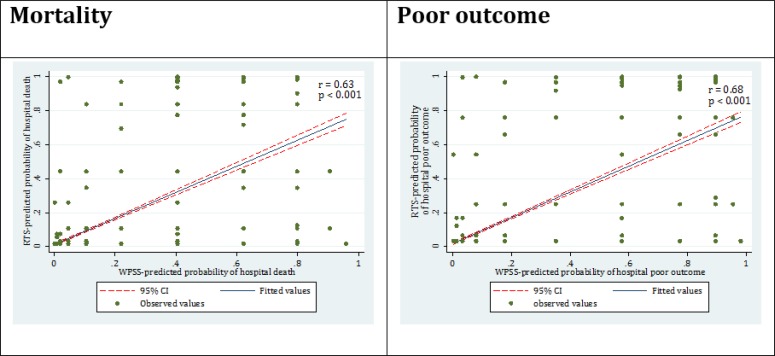
Concordance between revised trauma score (RTS) predicted and worthing physiological scoring system (WPSS) predicted percent of mortality and poor outcome

There was good concordance between RTS and WPSS models in prediction of mortality (r=0.63; p <0.001) and poor outcome (r=0.68; p <0.001) (Figure 3).

## Discussion

Classifying the severity of trauma in emergency settings is a challenging issue for the physicians. Scoring system can help to diagnosis of high risk patient. However, each scoring systems have specific advantages and limitations. The present study compared the two physiologic scoring systems of RTS and WPSS and found that the value of WPSS model in predicting mortality and occurrence of severe disabilities in trauma patients in higher than that of the RTS model. 

Although the RTS model involves simple criteria for estimating the severity of injuries, its prognostic value is at a moderate level. An acceptable scoring system for prediction of an outcome should have a high screening value along with a high sensitivity. The sensitivity of RTS model in prediction of mortality and poor outcome were 71.54% and 61.93%, respectively, while similar figures for the WPSS model were found to be 82.95% and 87.8%. Despite the greater number of variables included in the WPSS model compared to RTS model, its application is easy (11). WPSS is a physiologic scoring system which incorporates the respiratory rate, pulse rate, body temperature, arterial oxygen saturation and the level of consciousness. These factors can be easily assessed and are routinely evaluated in the emergency departments. The only factor that is not precisely measured in the emergency settings is the body temperature. In the busy hours of an emergency department, physicians or nurses might not pay adequate attention to accurate measuring of the patients’ body temperature, while assessment of this factor plays an important role in predicting the outcome of patients. Therefore, it is suggested that more attention be paid to the body temperature as a physiologic factor in patients referring to emergency departments. 

**Table 1:** Baseline characteristics of studied patients

Variable	Value
**Age (year)**	39.50 ± 17.27
**Gender **	
Male	1623 (75.56)
Female	525 (24.44)
**Trauma mechanism **	
Motorcycle accident	591 (27.51)
Car rider accident	518 (24.12)
Pedestrian	378 (17.60)
Falls more than 3 meters	152 (7.08)
Falls less than 3 meters	201 (9.36)
Other	308 (14.34)
**GCS **	14.4 ± 2.19
**HR (beat/minute)**	87.60 ± 15.63
**SBP (mmHg)**	115.38 ± 15.36
**DBP (mmHg)**	73.49 ± 10.07
**O2 saturation**	94.78 ± 5.80
**Temperature (Celsius)**	36.81 ± 0.90
**RR (n/minutr)**	16.46 ± 6.15
**Outcome **	
Good recovery	1630 (75.88)
Moderate disability	342 (15.92)
Sever disability	53 (2.47)
Death	123 (5.73)

Data were presented as mean ± standard deviation or number and percentage, GCS: Glasgow coma scale; HR: heart rate; SBP: systolic blood pressure; DBP: diastolic blood pressure; O_2_ saturation: Arterial oxygen saturation; RR: respiratory rate.

**Table 2:** Screening performance characteristics of revised trauma score (RTS) and worthing physiological scoring system (WPSS) in prediction of mortality and poor outcome

**Characteristics**	**Mortality**		**Poor outcome**	
	**RTS**	**WPSS**	**RTS**	**WPSS**
True positive	88	108	109	146
True negative	1972	1690	1940	1675
False positive	53	335	32	297
False negative	35	15	67	30
Sensitivity	71.54 (62.59-79.13)	87.80 (80.38-92.78)	61.93 (54.29-69.05)	82.95 (76.40-88.03)
Specificity	97.38 (96.56-98.01)	83.45 (81.75-85.04)	98.38 (97.69-98.87)	84.94 (83.27-86.47)
Positive LR	27.34 (20.49-36.46)	5.31 (4.72-5.97)	38.16 (28.56-54.85)	5.51 (4.86-6.24)
Negative LR	0.29 (0.22-0.39)	0.15 (0.09-0.24)	0.39 (0.32-0.47)	0.20 (0.14-0.28)

Data are presented as estimated value and 95% confidence interval. LR: Likelihood ratio.

**Table 3:** Overall performance of revised trauma score (RTS) and worthing physiological scoring system (WPSS) in prediction of in-hospital mortality and poor outcome

Characteristics	Mortality		Poor outcome	
	RTS	WPSS	RTS	WPSS
Brier score	0.026	0.031	0.038	0.045
Scaled reliability	<0.0001	0.0003	<0.0001	0.001

Few studies have assessed the prognostic value of WPSS for patients’ mortality. The findings of the present survey is congruent with the results of the study conducted by Duckitt et al. which has shown that the WPSS model is a better index for predicting patients’ mortality compared to the early-warning scoring system (11). Ha et al. also reported that both rapid emergency medicine score and WPSS have good prognostic values for mortality of patients in the emergency department, with the latter slightly superior to the former scoring system (23). Similarly, Brabrand et al. referred to the WPSS model as a scoring system with acceptable discriminatory power and calibration in predicting patients’ mortality (24). In this regard, it seems that the WPSS model can be used as a screening tool for classifying trauma patients in the emergency departments. 

The large sample size of the present study and its multi-center setting could be considered as the strengths of this survey warranting its power. Moreover, the results of this study can be generalized to the whole Iranian population since patients were included from emergency departments located in five different cities of Tehran, Ilam, Jahrom, Tabriz and Urmia. 

***Limitations***

The findings might be subject to selection bias due to the convenience sampling method used for inclusion of patients. Another factor that might have confounded the results of this survey was the probably inaccurate measurement of the patients’ axillary body temperature in the overcrowded emergency departments. 

## Conclusion:

The findings showed a higher prognostic value for the WPSS model in predicting mortality and severe disabilities in trauma patients compared to the RTS model. Both models had good overall performance in prediction of mortality and poor outcome. 
